# Perceptions of hearing loss and hearing technology among the general public and healthcare providers: a scoping review

**DOI:** 10.1136/bmjph-2024-001187

**Published:** 2024-10-15

**Authors:** Lauren K Dillard, Pallavi Mishra, Carolina M Der, Shelly Chadha

**Affiliations:** 1 Department of Otolaryngology Head & Neck Surgery, Medical University of South Carolina, Charleston, South Carolina, USA; 2 Department of Noncommunicable Diseases, World Health Organization, Geneva, Switzerland

**Keywords:** Public Health, Community Health, Social Medicine, education

## Abstract

**ABSTRACT:**

**Objectives:**

Synthesise literature related to perceptions of hearing loss and hearing technology (hearing aids, cochlear implants) among the general public and healthcare providers who do not specialise in ear and hearing care.

**Design:**

Scoping review.

**Data sources:**

We searched three databases (PubMed, Scopus, Ovid Medline) in August 2023 for peer-reviewed articles from inception to 2023.

**Eligibility criteria:**

Peer-reviewed articles or grey literature published in English or Spanish and that were observational or mixed methods were eligible for inclusion. Studies were included if they presented results from original research focused on perceptions of hearing loss and/or hearing technology among the general public and/or healthcare providers who do not specialise in ear and hearing care.

**Data extraction and synthesis:**

Two reviewers extracted and verified study data, which are synthesised in tables and in the text.

**Results:**

Twenty-nine peer-reviewed articles were included, 21 of which focused on samples of the general public and 8 on samples of healthcare providers. Perceptions about persons with hearing loss and the use of hearing technology are varied but often negative. The general public and healthcare providers often perceive hearing loss as less serious than other health conditions, including other sensory impairments. In many settings worldwide, the perception that non-biomedical factors, such as curses or evil spirits, can cause hearing loss is common. Importantly, studies showed training that aimed to raise awareness about hearing loss might improve negative or inaccurate perceptions of hearing loss and hearing technology.

**Conclusions:**

Perceptions of hearing loss and hearing technology are varied but often misinformed and negative. Such information could inform initiatives to raise awareness and change behaviours guided by those perceptions. Ultimately, changing the public’s and healthcare providers’ perceptions of hearing loss could encourage individuals with hearing loss to seek hearing care in a timely and appropriate manner.

**WHAT IS ALREADY KNOWN ON THIS TOPIC**

- Hearing loss and the use of hearing technology are stigmatised, but little is known about general attitudes or perceptions towards persons with hearing loss and/or use of hearing technology among the general public and healthcare providers who do not specialise in ear and hearing care. Negative or stigmatised attitudes or perceptions could cause avoidable delays in hearing loss diagnosis and rehabilitation.

**WHAT THIS STUDY ADDS**

- The evidence collated in this scoping review shows that perceptions of hearing loss and hearing technology are varied but often misinformed and negative.

**HOW THIS STUDY MIGHT AFFECT RESEARCH, PRACTICE OR POLICY**

- Study findings can be leveraged to inform initiatives to raise awareness and to change behaviours informed by prevalent misperceptions regarding hearing loss and/or hearing technology. Such initiatives could modify public perceptions of hearing loss and ultimately, encourage individuals with hearing loss to seek hearing care in a timely and appropriate manner.

## Introduction

 The WHO estimates over 430 million people worldwide have disabling hearing loss, and that this number will rise to 700 million by the year 2050 if prevention is not prioritised.^1^ Hearing loss affects individuals across the lifespan and has important impacts on individuals and society.^2^,^8^ It has been associated with poorer language development and educational attainment in children, and poorer quality of life, poorer employment prospects, and serious comorbid conditions such as cognitive decline in adults.^2^,^7^ The annual global costs of hearing loss are estimated to reach nearly US$1 trillion.^8^ While hearing loss impacts people worldwide, its negative impacts are exacerbated for those in low- and middle-income countries given limited access to hearing care, and limited awareness of hearing loss.^1^

Because hearing loss is a common and impactful health condition worldwide, it is important to understand how individuals perceive it. Extensive research, including systematic reviews, suggests hearing loss and hearing technology are stigmatised.^9^,^12^ However, social perceptions, which capture individual and population-level attitudes and mindsets towards hearing loss and/or hearing technology and could be positive, neutral or negative (including stigma), are not well described. In this study, we use the terms *perceptions* to understand this concept. This approach, rather than focusing only on stigma, facilitates a holistic understanding on how hearing loss and/or hearing technology is perceived.^13^

Perceptions of hearing loss/hearing technology, including negative (stigmatised) attitudes, can be perpetuated among the general public and could influence the uptake and utilisation of hearing technology.^14 15^ Furthermore, healthcare providers influence perceptions and behaviours of the persons they treat.^16 17^ This is important because individuals with hearing loss often first seek medical advice related to hearing loss from primary care.^17^ Understanding perceptions of the general public and healthcare providers could help comprehend and potentially, modify the behaviours informed by such perceptions.

Therefore, the objective of this systematically conducted scoping review was to synthesise literature related to perceptions of hearing loss and hearing technology among persons in the general public, and among healthcare providers who do not specialise in ear and hearing care.

## Methods

This scoping review was reported using the Preferred Reporting Items for Systematic Reviews and Meta-Analyses guidelines for scoping reviews.^18^ The protocol was registered with Open Science Framework (registration: osf.io/tkgeb). Given the nature of this review, a critical appraisal of individual sources of evidence was not conducted.

### Eligibility criteria

Peer-reviewed articles or grey literature published in English or Spanish and that were observational or mixed methods were eligible for inclusion. The language criterion was determined based on the languages the study team speaks fluently. Articles published in English were identified through a systematic search and articles in Spanish were identified through hand-searching methods only.

No eligibility restrictions were placed on publication year. Studies were included if they presented results from original research focusing on perceptions of hearing loss and/or hearing technology, defined as hearing aids (any type) or cochlear implants, among the general public and/or healthcare providers who do not specialise in ear and hearing care. Studies were excluded if (a) the sample consisted only of individuals with hearing loss, or parents of children with hearing loss (b) they focused only on awareness or knowledge, rather than perceptions or (c) they focused only on stigma of hearing loss and/or hearing technology, given that several systematic reviews on stigma exist.^9 11 12^

### Information sources and search

To identify articles published in English, the electronic databases PubMed, Scopus and Ovid Medline were searched on 28 August 2023. Pilot searches confirmed the sensitivity and specificity of search terms. Search strings are in online supplemental file 1. Reference lists of all included studies were searched to identify additional articles.

We searched grey literature to identify high-quality, relevant evidence that was not available in published, peer-reviewed articles. Grey literature sources, published in English, including white papers, newsletters, reports or proceedings were identified by using the keywords (or derivations of) hearing loss, deafness, perceptions and attitudes. We also searched Google Scholar to detect grey literature sources, such as published abstracts or conference papers, theses and dissertations.

Search terms were translated into Spanish by a native Spanish speaker. Articles and grey literature were identified in Google Scholar using handsearching methods.

### Selection of sources of evidence

Titles and abstracts of manuscripts were screened by a single reviewer. Next, full text articles in English were screened by two reviewers and differences were reconciled via discussion of manuscripts. A single, native Spanish-speaking reviewer screened Spanish articles, as only one team member was fluent in Spanish. The entire study team verified inclusion and exclusion decisions for all articles by discussing reasons for including/excluding each article after the full-text screen.

### Data charting process and data items

Before conducting this review, data collection tables were developed and piloted to extract data from several relevant articles. For studies published in English, a single reviewer extracted study data, which a second reviewer verified. For studies published in Spanish, a single reviewer extracted data.

Data collection tables included details on (a) meta study information (eg, author, year, journal), (b) study and sample characteristics (eg, study design, participant age, location (country)) and (c) key results related to perceptions of hearing loss and/or hearing technology. The charted data are synthesised in the tables presented in the Results section.

The authors of the included articles use terms including hearing loss and D/deaf. These terms can capture nuances in culture, communication and severity of hearing loss. Because authors of individual articles rarely define those attributes, we synthesise results using the same terms used by authors of a given article.

### Patient and public involvement

Patients or the public were not involved in the design, conduct or reporting of this scoping review.

## Results

A total of 303 non-duplicate peer-reviewed citations were identified, with 29 articles included after the final review. Online supplemental figure 1 shows the study selection process.^19^ The studies included participants from 14 countries, from African (4 studies), American (9 studies), European (8 studies), South-East Asian (8 studies) and Western Pacific (2 studies) regions. 28 studies were published in English and 1 in Spanish.

The Results section is organised as follows. First, we present results related to perceptions of hearing loss and/or hearing technology among the general public, and then among healthcare providers who do not specialise in ear and hearing care. Finally, we present relevant findings from grey literature sources.

### Perceptions of hearing loss and/or hearing technology among the general public

Tables1^3^ display characteristics of the 21 studies focused on perceptions of hearing loss and/or hearing technology among the general public.^20^,^40^ Twenty studies were conducted in samples of adults and 1 in school-aged children.

**Table 1:** Overview of studies in samples of the general public focused on perceptions of hearing loss

First author (year)country	Aims	Study design	Participant details	Primary results related to hearing
**Importance of hearing losscompared withother conditions**				
Bray *et al*^20^ (2019)USA	Examine perceptions of what it would be like to live with an impairment or disability.	Cross-sectional pilot surveyPerceived impacts of disability measured by EQ-VAS	151 undergraduate students.Mean age 20 (SD 3.0) years	Hearing impairment was perceived to have lowest impact on health status of the following: hearing, visual, mobility impairment.
Scott *et al*^21^ (2016)USA	Evaluate attitudes and awareness of Americans towards vision loss and eye-health.	Cross-sectional survey	2044 members of the general public.Mean age 46.2 (range 18–99) years	Loss of hearing was ranked as having the least effect on day-to-day life, following loss of eyesight, memory, limb condition and speech, and the least important in terms of ‘worst conditions’, following blindness, Alzheimer’s disease, cancer, AIDS/HIV, loss of limb, heart disease and arthritis.
**Perceptions of occupational hearing loss**				
Hétu *et al*^34^ (1994)*Canada	Develop a questionnaire that characterises attitudes towards individuals with occupational hearing loss.	Cross-sectional survey (development and testing)	280 workers in a noisy occupational setting, and who employ workers with noise-induced hearing loss.Mean age 42.6 (9.4) years57% had audiometric hearing loss; 66% had perceived hearing loss	Attitudes towards coworkers with noise exposure were favourable but may result from social desirability bias. Most individuals did not adopt behaviour that would improve communication with coworkers with hearing loss.
Hétu *et al*^35^ (1994)*Canada	Identify workers’ perceptions of coworkers with occupational hearing loss.	Cross-sectionalFocus group interviews	35 workers.Age range 35 (SD 7.1) to 49 (SD 6.3) years†	Workers were unaware of the manifestations and consequences of hearing loss. Coworkers with hearing loss were stigmatised.
**School children’s perception of hearing loss**				
Cambra^36^(2005)Spain	Evaluate school children’s perceptions of hearing loss and the integration of deaf students in the classroom.	Cross-sectional survey	73 students who shared their classroom with a classmate with hearing loss.Age range 10–14 years	Students held stereotyped attitudes towards classmates with hearing loss.Students were unable to identify how they were similar/different to children who had hearing loss.Students had positive perceptions towards integrating children with hearing loss into mainstream classrooms.

* Studies were published in the 1990s so could hold limited relevance to current perceptions of hearing loss.

† Mean (SD) age is presented for four focus groups, separately, and areis presented as a range (lowest to highest mean age).

EQ-VAS, EuroQol-Visual Analogue Scales

**Table 2:** Overview of studies in samples of the general public focused on perceived causes of hearing loss

First author (year)country	Aims	Study design	Participant details	Primary results related to hearing
**Perceived non-biomedical causes of hearing loss**				
de Andrade and Ross^22^ (2009)*South Africa	Investigate the beliefs and practices of South African traditional healers on hearing impairment.	Cross-sectional, mixed-methods consisting of interviews	15 black South African traditional healers across 6 ethnic groups.Age range 21–70 years	All traditional healers included in the study report being consulted for hearing and/or ear-related conditions.Common perceptions on the non-biomedical causes of hearing problems were ancestors, bewitchment and blood impurities. Hearing problems were diagnosed and treated using several methods.
Kaspar *et al*^23^ (2017)Solomon Islands	Investigate parental knowledgeand attitudes towards hearing loss and childhood services in the Solomon Islands.	Cross-sectional semi-structured interview	153 parents attending Child Welfare Clinics.39% aged 20–29, 42% aged 30–39, 9% aged 40–49, 1% aged 50–59 and 1% aged 60–69 years	56% believed curses may cause hearing loss.22% believed evil spirits could cause hearing loss. Individuals who were older and male were more likely to hold this belief.
Govender and Khan^24^ (2017)South Africa	Describe knowledge of mothers on risk factors of infant hearing loss and availability of services.	Cross-sectional survey	102 mothers attending public sector antenatal clinics.Mean age 24 (range 18–45) years	56% believed ancestral curses could cause hearing loss.61% believed angry ancestors could cause hearing loss.
Swanepoel and Almec^25^ (2009)South Africa	Investigate maternal knowledge and attitudes towards hearing loss.	Cross-sectional, face-to-face survey	100 mothers attending regional immunisation clinic as part of public healthcare system.Aged >18 years	44% believed blood impurities could cause hearing loss.29% believed bewitchment could cause hearing loss.
Seguya *et al*^26^ (2021)Uganda	Explore maternal knowledge on causes of infant hearing loss.	Cross-sectional, semi-structured questionnaire	401 mothers of infants who were recruited from the postnatal or paediatric wards, or postnatal or paediatric outpatient clinics.Mean age 25 (SD 5.6) years	57% believed angry ancestors could cause hearing loss.60% believed witchcraft could cause hearing loss.
Aljabri *et al*^27^ (2019)Saudi Arabia	Investigate parental knowledge about risk factors for hearing loss.	Cross-sectional survey	Parents of children attending paediatric clinics of hospital	46% believed curses could cause hearing loss.17% believed evil spirits could cause hearing loss.
Aljuaid *et al*^28^ (2021)Saudi Arabia	Assess parental knowledge about risks and causes of hearing loss.	Cross-sectional survey	464 parents living in specific city. 83% were mothers.Aged ≥20 years	21% believed curses could cause hearing loss.31% believed evil spirits could cause hearing loss.
Alsudays *et al*^29^ (2020)Saudi Arabia	Evaluate parents’ knowledge attitudes regarding childhood hearing loss.	Cross-sectional survey	243 parents (57% mothers) attending paediatric and ENT clinics.20% aged 21–30, 35% aged 31–40, 28% aged 41–50, 11% aged 51–60 and 6% aged >60 years	58% and 52% of fathers and mothers believed curses could cause hearing loss.41% and 38% of fathers and mothers believed evil spirits could cause hearing loss.
Elrefaie *et al*^30^ (2022)Saudi Arabia	Assess caregivers’ and parents’ awareness and knowledge about hearing loss.	Cross-sectional survey	1618 caregivers and parents (72% female).4% aged <20, 29% aged 20–30, 28% aged 31–40, 27% aged 41–50, 10% aged 51–60 and 2% aged >60 years	13% believed evil spirits could cause hearing loss.49% believed envy could cause hearing loss.
Yoshita *et al*^31^ (2023)India	Evaluate knowledge and attitudes of parents and caregivers regarding childhood hearing loss.	Cross-sectional interview	314 mothers, fathers, and caregivers in urban and rural populations.Mean age 29.6 (SD 6.6) years (range 19–63)	18%, 14% and 12% of fathers, mothers and caregivers in urban populations believed curses could cause hearing loss.9%, 16% and 18% of fathers, mothers and caregivers in rural populations believed curses could cause hearing loss.
Rajagopalan *et al*^32^ (2014)India	Investigate opinions of grandmothers of newborns on hearing loss.	Cross-sectional survey	100 grandmothers of newborns, recruited from postnatal ward	19% believed bewitchment could cause hearing loss.24% believed ancestral sins could cause hearing loss.
Kaspar *et al*^33^ (2023)Samoa	Assess knowledge and attitudes to childhood hearing loss and hearing services in Samoa.	Cross-sectional semi-structured interview	150 Samoan female caregivers residing in both urban and rural areas.Mean age 31.8 (SD 8.7) years	40% believed curses could cause hearing loss.9% believed evil spirits could cause hearing loss.

* Traditional healers were classified under the section of samples of the general public, rather than healthcare providers, because most traditional healers do not have formalizedformalised medical training.

ENTear-nose-throatEQ-VASEuroQol-Visual Analogue Scales

**Table 3:** Overview of studies in samples of the general public focused on social representation of hearing loss and/or hearing technology

First author (year)country	Aims	Study design	Participant details	Primary results related to hearing
**Social representation of hearing loss**				
Manchaiah *et al*^37^ (2015)India, Iran, Portugal, UK	Understand social representation of hearing loss and how it varies by country.	Cross-sectional survey.Free association task: participants reported words or phrases that came to mind when thinking of hearing loss	404 members of the general public.Mean age 41 (SD 17) years	The five most common categories of free association task responses were: assessment and management, causes of hearing loss, communication difficulties, disability, and hearing ability or disability.Perceptions of hearing loss were neutral, positive and negative.
Germundsson *et al*^38^ (2018)India, Iran, Portugal, UK	Determine how social representation of hearing loss varies by country and demographic factors (age, employment status, education).	Cross-sectional survey.Free association task	404 members of the general public.Mean age 41 (SD 17) years	Social representation of hearing loss is largely robust to age and employment status but differs by education and country of origin.
**Social representation of hearing aids**				
Manchaiah *et al*^39^ (2015)India, Iran, Portugal, UK	Understand social representation of hearing aids.	Cross-sectional survey.Free association task	404 members of the general public.Mean age 41 (SD 17) years	The five most common categories of free association task responses were: hearing instruments; appearance and design; improved hearing and communication; assessment and management; cost.Perceptions of hearing aids were neutral, positive and negative.
Manchaiah *et al*^40^ (2018)India, Iran, Portugal, UK	Determine if the social representation of hearing aids varies by country and/or demographic factors.	Cross-sectional survey.Free association task	404 members of the general public.Mean age 41 (SD 17) years	Perceptions of hearing aids varied by country and demographic factors (education, occupation).

These studies are organised by the following categories: (a) importance of hearing loss compared with other conditions (table 1), (b) perceived non-biomedical causes of hearing loss (table 2), (c) perceptions of occupational hearing loss (table 1), (d) school children’s perception of hearing loss (table 1), (e) social representation of hearing loss and (f) social representation of hearing aids (table 3).

#### Importance of hearing loss compared with other conditions

Two survey-based studies evaluated perceived impacts of hearing loss compared with other conditions/impairments (table 1).^20 21^ In a pilot study, US students estimated their perceived health status on the EuroQol-Visual Analogue Scales tool for 12 states of impairment, capturing visual, hearing and mobility impairment across four states: moderate, moderate with adaptation (eg, use of eyeglasses, hearing aid or mobility aid), severe and severe with adaptation.^20^ Hearing (vs visual and mobility) impairment was estimated to be least impactful on health status across all states of impairment. The difference between perceived impacts of hearing and vision impairment was more similar for severe (vs moderate) states of impairment, suggesting the perceived impacts of hearing impairment vary by severity.^20^

In another survey-based study of the general USA public on sensory impairment and general health, participants ranked loss of hearing as the least impactful on daily life, compared with loss of memory, eyesight, speech or limb function.^21^ Less than 5% of participants ranked loss of hearing as the most impactful, which contrasts with approximately 50% who answered loss of vision. When asked which condition was the worst that could happen, deafness ranked the lowest (compared with arthritis, blindness, heart disease, cancer, AIDS/HIV, loss of limb, Alzheimer’s disease). Approximately 2% of participants reported deafness, whereas approximately 20% and 18% ranked blindness and Alzheimer’s disease (the highest-ranking conditions), respectively, as the worst that could happen.^21^

#### Perceived non-biomedical causes of hearing loss

Twelve studies focused on perceived non-biomedical causes of hearing loss (table 2).^22^,^33^ One study reported South African traditional healers’ perceptions on the causes, diagnosis and treatment of hearing problems.^22^ All healers reported being consulted for ear and hearing conditions by persons from across the age range, mostly related to hearing loss/deafness, ear discharge and ear pain. Most believed hearing loss could be caused by ancestors, and others thought it could be caused by bewitchment, blood impurities, disturbance of the inner spirit or because the patient was in the wrong place, which refers to a concept in which a person is in a place believed to be contaminated. Some acknowledged medical causes, including noise, poor aural hygiene and ageing. They diagnosed hearing problems through divinatory throwing of the bones, looking in the ear, and feeling the patient’s pain and knowing what is wrong. Treatments included plant-based medicines and animal-based products and prayer.^22^

The remaining studies on perceived non-biomedical causes of hearing loss were conducted in parents,^23^,^31^ grandparents^32^ and/or caregivers.^30 31 33^ Across studies, 9%–58% of participants believed curses,^23 24 27 28 31 33^ and 9%–41% believed evil spirits could cause hearing loss.^2327^,^30 33^ Similarly, 57%–61% of participants believed angry ancestors,^24 27^ and 24% believed ancestral sins,^32^ could cause hearing loss. Participants also believed bewitchment (19%–29%),^25 32^ witchcraft (60%),^26^ blood impurities (44%)^25^ and envy (49%),^30^ could cause hearing loss. Many participants reported not knowing whether non-biomedical factors could cause hearing loss.^23^,^2932 33^ Beliefs varied by country and region. One study reported older individuals were more likely to believe in evil spirits as a cause of hearing loss,^23^ and some studies reported men were more likely to believe in non-biomedical causes of hearing loss,^23 29 31^ although demographic differences in beliefs were not consistent across all studies.^31 33^

#### Perceptions of occupational hearing loss

Two studies focused on workers’ perceptions of their coworkers with occupational hearing loss (table 1).^34 35^ In the first study, authors concluded supervisors’ attitudes towards workers with noise-induced hearing loss were generally favourable but that responses were likely influenced by social desirability bias. Authors also concluded most supervisors did not adopt behaviour to improve communication with individuals with hearing loss.^34^ In the second study, workers without hearing loss were interviewed to determine their perceptions of colleagues with occupational hearing loss. Key results were that workers were unaware of the negative impacts of hearing loss on their colleagues, and coworkers with hearing loss were stigmatised.^35^

#### School children’s perception of hearing loss

One study surveyed school-aged children (without hearing loss) who had a classmate with hearing loss about their perceptions of hearing loss and the integration of deaf students in the classroom (table 1).^36^ The author concluded students used stereotyped concepts to define deafness and had problems understanding how they are similar and different to children with hearing loss. The students showed positive perceptions towards integrating children with hearing loss into mainstream classrooms.^36^

#### Social representation of hearing loss

Two survey-based studies, using the same data, focused on the social representation of hearing loss among individuals in India, Iran, Portugal and the UK, and how it may vary by country,^37^ and demographic factors (table 3).^38^ Social representation can be defined as knowledge and information common to groups, and is used to guide communication and organise behaviour.^37 41 42^

Participants completed a free association task, in which they (a) wrote the first 4–5 words or phrases that came to mind when thinking about hearing loss, then (b) indicated whether those words or phrases had positive, neutral or negative connotations. The most common response themes were: assessment and management, causes of hearing loss, communication difficulties, disability, hearing ability or disability, hearing instruments, negative mental state, attitudes of others, and sound and acoustics of the environment.^37 38^

Among respondents from all countries, approximately 25% and 17% of responses related to hearing loss were assigned positive or neutral connotations, respectively, whereas approximately 55% of responses were assigned negative connotations. There were significant differences across countries. For example, in India, 40%, 20% and 40% of responses had positive, neutral and negative connotations, respectively. In the UK, 15%, 20% and 65% of responses had positive, neutral and negative connotations, respectively.^37^ Authors concluded negative associations, often linked to aspects of disability, were most common. Authors also concluded some people perceive hearing loss positively, and there are cross-cultural differences in the social representation of hearing loss.^37^

Another study aimed to determine associations of demographic factors, including age, employment status, education and country of origin, with the social representation of hearing loss.^38^ Authors used cluster analysis to group survey responses into the following five clusters: (1) individual aspects, (2) aetiology, (3) the surrounding society, (4) limitations and (5) exposed. Age was associated with two clusters (1, 3) and employment status was associated with one (1). Education (1, 3, 4, 5) and country of origin (2, 3, 4, 5) were associated with four of the five clusters. Authors concluded the social representation of hearing loss was robust to some demographic differences, including age and employment status, but that some factors, including education and country of origin, likely impact perceptions of hearing loss.^38^

#### Social representation of hearing aids

Two studies focused on the social representation of hearing aids in the same sample as the studies above and used similar analytic methods (table 3).^37^,^40^ The most common response themes for hearing aids were: hearing instruments, appearance and design, improved hearing and communication, assessment and management, cost, disability, improved life condition, ageing and beneficial.^39 40^

Among respondents from the four countries, approximately 42% and 18% of responses related to hearing aids had positive or neutral connotations, respectively, whereas approximately 40% of responses had negative connotations. There were no significant differences in these percentages across the countries, suggesting there may be cultural similarities in the perceptions of hearing aids.^39^ While 40% of responses had negative connotations, this is substantially lower than the 55% of responses with negative connotations of words/phrases related to hearing loss.^37 39^

In another article, authors used qualitative content analysis and quantitative cluster analysis, identifying five main clusters: (1) positive attitude, (2) external factors, (3) hearing aid use and satisfaction, (4) aetiology and (5) benefits and limitations of technology. Demographic factors influenced patterns of responses. Education was related to three (2, 4, 5), occupation was related to one (4) and country was related to all five clusters (1, 2, 3, 4, 5). Authors concluded cross-cultural and demographic differences likely influence perceptions of hearing aids.^40^

### Perceptions of hearing loss and/or hearing technology among healthcare providers

Tables4  5 display characteristics of the eight studies focused on perceptions of hearing loss and/or hearing technology among healthcare providers who do not specialise in ear and hearing care.^43^,^50^ All studies were conducted in samples of adults.

**Table 4:** Overview of studies in samples of healthcare providers focused on perceptions of hearing loss and/or hearing technology, including hearing aids and cochlear implants

First author (year)country	Purpose	Study design	Participant details	Primary results related to hearing
**Perceived importance or impacts of hearing loss**				
Sydlowski *et al*^43^ (2022)USA	Characterise perceptions, awareness and literacy surrounding hearing loss among primary healthcare professionals.	Cross-sectional survey	406 (205 primary care physicians; 201 nurse practitioners or physician assistants).Age data not reported	Compared with other conditions, providers found hearing loss as less important to address. Less than half of providers regularly recommended screening. 24% and 33% of providers did not think hearing loss was impactful for personal safety and overall health, respectively.
Ralston *et al*^44^ (1996)*USA	Compare physicians’ attitudes towards their deaf patients vs their patients in general.	Cross-sectional survey	165 (71 received questionnaire focused on deaf patients; 94 received questionnaire focused on their patients in general).Age data not reported	Physicians who received the questionnaire focused on deaf patients reported poorer attitudes towards their (deaf) patients.
Ebert and Heckerling^45^ (1995)*USA	Assess physicians’ knowledge and beliefs on communication with deaf people.	Cross-sectional survey	73 physicians at a university medical centre.Mean age 43.2 (SD 10.2) years	55% of physicians thought time/effort to provide services was slightly greater and 45% much greater (vs normal hearing).63.4% of providers knew they should use sign language interpreters to communicate with deaf patients. 32.4% thought it was appropriate to write back and forth with the patient and 4.2% thought the patient should lipread.
López-Vázquez *et al*^46^ (2009)Mexico	Evaluate attitudes and knowledge towards hearing loss among medical doctor residents.†	Cross-sectional survey	2727 medical doctors.Age data not reported	85% of participants did not think hearing loss could become extremely disabling.
Yerraguntla *et al*^47^ (2016)India	Evaluate attitudes and knowledge of paediatric hearing loss among three groups working in primary healthcare settings.	Cross-sectional survey	90 (30 general physicians; 30 medical interns in practice where speech and hearing services were available; 30 medical interns in practice where such services were not available).Age data not reported	Most participants believed that hearing is important for speech and language development. Agreement was higher among general physicians than medical interns.
**Perceptions of hearing technology**				
Reddy *et al*^48^ (2022)India	Assess knowledge, attitude and practices regarding cochlear implants among healthcare providers at a tertiary care academic institution.	Cross-sectional survey	100 general physicians.Age range 25–61 years	Most providers agreed that in a country like India, cochlear implantation was relevant for children (84%) but less so for adults (53%) who lose hearing in both ears.

* Studies were published in the 1990s so could hold limited relevance to current perceptions of hearing loss.

† 2.6% of the sample specializedspecialised in ear and hearing care (audiology or otolaryngology).

**Table 5:** Overview of studies focused on interventions to modify perceptions of hearing loss among healthcare providers

First author (year)country	Purpose	Study design	Participant details	Intervention description	How intervention success was measured	Primary results
Gilmore *et al*^49^ (2019)UK	Determine the impact of deaf awareness training on medical students’ attitudes towards and knowledge of deafness.	Mixed methods	59 (intervention group: 29; controls: 30)	Intervention group participated in a 12-week module, which included sign language classes, deaf awareness sessions and an optional examination.	Validated questionnaire measured attitudes and deafness before and after participation in module. Control group (who did not participate in module) answered questionnaire.Focus groups were conducted to learn about participants’ experiences and explore ways to incorporate deaf awareness training into curriculum.	Intervention group had more positive attitudes towards deafness (vs controls and pre-post).
Hoang *et al*^50^ (2021)USA	Determine the impact of Deaf community training programme on medical students’ perceptions, culture competency and knowledge.	Mixed methods	372 (intervention group: 25; control group of medical students: 211; control group of faculty: 30)	Intervention group participated in 2-year fellowship that included sign language classes, a 2-month Deaf culture immersion programme, and specialised workshops.	Survey was created by researchers and focused on: misperceptions of deafness, common difficulties experienced by Deaf persons in healthcare settings, errors commonly made by providers, and exposure to the Deaf community.Intervention and control groups completed survey.	Intervention group had higher knowledge score than both control groups.Intervention group could assess Deaf populations’ challenges with healthcare more holistically than both control groups.

The studies in this section are organised by the following categories: (a) perceived importance or impacts of hearing loss, (b) perceptions of hearing technology (table 4) and (c) interventions to modify perceptions of hearing loss among healthcare providers (table 5).

#### Perceived importance or impacts of hearing loss

Five studies evaluated perceived importance (vs other conditions), and/or impacts of hearing loss.^43^,^47^ In a survey of US healthcare providers, hearing loss was ranked the least important condition to address in patients 50 years and older, compared with Alzheimer’s disease, arthritis, asthma, cancer, chronic obstructive pulmonary disease, diabetes, heart disease, high blood pressure, obesity and vision loss.^43^ Only 46% of providers reported commonly discussing or recommending hearing screening, compared with 60% for vision screening. In terms of perceived impacts of hearing loss, 76% of providers thought hearing loss could impact personal safety, and only 67% thought it was important to overall health.^43^

Another study compared primary care physicians’ perceptions of their deaf patients versus their patients in general.^44^ One group completed a questionnaire on perceptions towards deaf patients, specifically, whereas the control group completed a questionnaire on perceptions towards all patients. Physicians reported greater difficulties understanding and maintaining conversations, feeling less comfortable and asking fewer questions to deaf patients. They reported deaf patients have difficulties understanding and asked for repetition, get frustrated easily, rely on interpreters, do not trust the physicians, and were less likely to understand diagnosis and treatment. All physicians surveyed lacked adequate knowledge of legal requirements for providing interpretation for deaf patients.^44^ Another study reported all surveyed physicians perceived the time and effort to provide services to deaf persons was much (45%) or slightly greater (55%) compared with persons without hearing loss.^45^ When asked about methods to communicate with deaf persons, 63.4% knew sign language interpreters should be available, 32.4% thought it was appropriate to write back and forth with deaf persons and 4.2% thought deaf persons should use lipreading to communicate with providers.^45^

Two studies surveyed medical providers on impacts and importance of hearing loss. In one study, few physician residents strongly agreed (2.7%) or agreed (11.9%) hearing loss is a condition that can become ‘extremely disabling’, whereas 1.8% neither agreed nor disagreed, 51.8% disagreed and 31.1% strongly disagreed.^46^ In another study on paediatric hearing loss, most general physicians agreed or strongly agreed (93%) hearing was important in the development of speech and language in children, but fewer medical interns (77%) responded similarly.^47^

#### Perceptions of hearing technology among healthcare providers

One study on perceptions of hearing loss and hearing technology among Indian healthcare providers showed 93% of providers reported good hearing ability is important for patients, and the remaining 7% thought hearing loss could be managed with minor adaptations.^48^ Regarding cochlear implants, 84% (the remaining 16% were unsure) reported cochlear implantation was relevant and important in a country like India for children with bilateral hearing loss. Fewer (53%) thought cochlear implantation was relevant and important for adults with bilateral hearing loss; 9% disagreed and 38% were unsure.^48^

#### Training or intervention studies

Two studies focused on trainings or interventions to improve perceptions and knowledge of medical students towards persons with deafness (table 5).^49 50^ One consisted of a 12-week training including sign language classes, deaf awareness sessions and an optional examination. Participants who underwent the training, versus those who did not, had more positive attitudes towards Deaf persons.^49^ The other study consisted of a 2-year fellowship with sign language classes, a 2-month deaf culture immersion programme and workshops.^50^ Compared with control groups of medical students who did not undergo the programme and faculty members, persons who underwent the fellowship had fewer misperceptions on deafness and Deaf culture, and greater knowledge on the difficulties experienced by Deaf patients and sign language interpretation. Persons who underwent the training (vs control groups) could holistically describe the difficulties with healthcare that Deaf people experience and were able to relate those difficulties to Deaf culture and community rather than only physiological differences between persons who do and do not have deafness.^50^ In both studies, participants ranked their participation in the programmes favourably.^49 50^

### Grey literature

Three grey literature sources were identified, all of which were theses or dissertations.^51^,^53^

### Perceptions of hearing loss and/or hearing technology among the general public

One study developed a questionnaire related to perception towards people with hearing loss in Singapore, which largely focused on abilities of persons with hearing loss. The questionnaire was administered to 312 university students. Based on averaged scores on the questionnaire, the author concludes participants generally perceived persons with hearing loss positively.^51^

Chadha reported on ear and hearing care knowledge, attitudes/perceptions, behaviours and practices in India. Data were obtained through questionnaires, focus groups and in-depth interviews.^52^ Key findings include that many parents and teachers believed hearing loss was untreatable. Most teachers believed deaf children could not be admitted to a regular school, and several believed deaf children could not learn to speak. Parents, teachers and students reported children with hearing loss could have difficulties in education, employment and performing daily activities. Parents, teachers and students believed in non-biomedical causes of hearing loss, reporting that children can be born deaf because of the mother’s exposure to sunlight during a solar eclipse, and/or from a curse of the Gods. Most parents thought children should live with hearing loss, rather than wear hearing aids, because use of hearing aids would tarnish the reputation of the family and cause their child to be different than others. Most teachers believed hearing aids are not beneficial, uncomfortable and stigmatised.^52^

### Perceptions of hearing loss and/or hearing technology among healthcare providers

Chadha also reported on attitudes/perceptions among primary and general practitioners and community health workers. Most healthcare providers were aware of treatment options for hearing loss, including hearing aids, speech therapy and cochlear implants. However, many community health workers did not know when or how hearing could be tested, and believed it was suitable to wait a few years to see if the child started speaking before sending the child for testing or intervention. Most healthcare providers knew hearing loss could impact speech development, education and employability. Some community health workers (but not other providers) believed in non-biomedical causes of hearing loss.^52^

A survey-based study of 96 primary care physicians in New York, USA assessed attitudes/perceptions towards hearing technology.^53^ Most physicians (84%) reported their patients believe hearing aids are a worthwhile investment. The majority agreed (54%) or somewhat agreed (25%) most persons with age-related hearing loss benefit from hearing aids (neutral: 10%; somewhat disagree: 9%; disagree 1%). When asked if the physicians themselves would purchase hearing aids if they had difficulty hearing, most agreed (54%) or somewhat agreed (22%), with slightly higher proportions reported if hearing aids were recommended by a hearing specialist (agree: 59%; somewhat agree: 30%). While most primary care physicians recognised benefits of hearing aids, few reported discussing hearing problems (always: 13%; usually: 18%; occasionally: 63%; never: 6%) or hearing aids with patients (always: 3%; usually: 4%; occasionally: 59%; never: 33%).^53^

## Discussion

This scoping review summarises evidence on perceptions of hearing loss and/or hearing technology among the general public and healthcare providers who do not specialise in ear and hearing care. Understanding these perceptions could inform initiatives to raise awareness about hearing loss, and to modify behaviours informed by misperceptions, thus reducing misperceptions towards individuals with hearing loss. This is important because such misperceptions may influence individuals’ decisions to seek hearing care and may ultimately cause unnecessary delays in hearing loss diagnosis and rehabilitative interventions.

This review aimed to synthesise evidence on perceptions of hearing loss and hearing technology. We excluded studies on stigma and hearing loss, as they have been comprehensively described in existing literature.^9^,^12^ Stigma can influence how individuals with hearing loss perceive themselves, which could lead to individuals not appropriately addressing their hearing loss.^9^ There is extensive research on the ‘hearing aid effect’, in which individuals without hearing loss assign negative attributes towards hearing aid users, thinking of them as, for example, less intelligent, less athletic and less trustworthy.^54^,^56^ While some studies suggest the hearing aid effect is less pervasive in recent years,^55 56^ others suggest it persists today.^57 58^

### Perceptions of hearing loss and/or hearing technology among the general public

Studies in this review suggested the general public perceives hearing loss as less serious than other health conditions, including other sensory impairments. This may be because hearing loss is often thought of as a normal part of ageing, rather than an urgent health condition that needs to be addressed.^10^ Yet, unaddressed hearing loss is impactful on individuals across the lifespan, including on language development and learning among children, and activities of daily living, employability, quality of life and cognitive function among adults.^2^,^7^ The public perception that hearing loss is not an important health condition could deter individuals from seeking hearing care, leading to delayed diagnosis and intervention.^59^

This review highlighted the common belief (particularly in low- and middle-income countries) that non-biomedical factors, such as curses or evil spirits, can cause hearing loss.^22^,^33^ These beliefs could cause negative attitudes towards individuals with hearing loss and their families, potentially leading to them not addressing the issue.^52^ They could also influence expectations related to hearing loss; for example, individuals may believe permanent conditions, such as hearing loss, can be cured.^60^ Similar non-biomedical beliefs, and their impacts on behaviours, have been described for other conditions or disabilities.^60^,^63^

In this review, several studies showed people hold negative perceptions of their peers with hearing loss.^34^,^36^ Key themes were that people (a) were unaware of impacts of hearing loss, (b) did not change their behaviours to accommodate peers with hearing loss and (c) held stereotyped attitudes towards peers with hearing loss.^34^,^36^

In studies on the social representation of hearing loss and hearing aids, most respondents associated the term hearing loss with negative connotations, and on average, individuals viewed hearing aids more positively than they viewed hearing loss.^37^,^40^ A substantial proportion of respondents viewed hearing loss and hearing aids either neutrally or positively, indicating variation in how they are perceived. This finding is consistent with other studies in this review showing some attitudes towards individuals with hearing loss were positive.^34^,^36^ Importantly, few studies explicitly explore positive/neutral perceptions towards hearing loss. There were cross-cultural and regional differences in the social representation of hearing loss, for example, individuals in some countries were more likely to perceive hearing loss or hearing aids positively.^38 40^ Understanding cross-cultural differences, and other factors related to perceptions of hearing loss, could inform tailored messaging to improve awareness of hearing loss.

### Perceptions of hearing loss and/or hearing technology among healthcare providers

Like findings from studies of the general public, some studies showed healthcare providers perceive hearing loss as less serious than other conditions, including other sensory impairments.^43 46^ These perceptions likely influence perceptions of the persons they treat. If providers do not perceive hearing loss as a health priority and do not regularly discuss hearing loss or hearing aids with their patients,^53^ persons seeking advice from their medical providers are probably less likely to view their hearing loss as important. This could perpetuate the misperception that hearing loss is not important for health and well-being.^43^ One study showed some community health workers in India believed non-biomedical factors could cause hearing loss.^52^ Although other studies did not report on this, similar perceptions among healthcare providers are likely common where non-biomedical beliefs are widespread. Some studies showed healthcare providers had limited awareness of hearing aids and cochlear implants,^48 52 53^ and hearing testing and technology were rarely discussed with patients.^48 53^ These perceptions, awareness and behaviours likely impact the perceptions and health-seeking behaviours of the persons they treat.

Furthermore, this review showed some providers have negative attitudes towards their patients with hearing loss and find it burdensome to interact with them.^44 45^ Although those studies were conducted in the 1990s, recent studies show communication barriers between persons with hearing loss and healthcare providers persist, and most healthcare providers are aware they do not provide appropriate accommodation for patients with hearing loss.^64 65^

Importantly, training could improve medical providers’ knowledge, awareness and perceptions towards individuals with hearing loss, suggesting it is possible to modify misperceptions of hearing loss and/or hearing technology.^49 50^ Other research shows healthcare providers often realise their hearing-related knowledge is limited, but that they are interested in receiving training related to hearing loss and hearing care.^66 67^ Such training could be valuable to change perceptions towards hearing loss and hearing technology given individuals often first seek hearing-related advice from general physicians.^17 68^

### Synthesis

In summary, hearing loss and hearing technology are often, but not always, misperceived among the general public and healthcare providers who do not specialise in ear and hearing care. These misperceptions likely contribute to people denying their hearing loss and avoiding or delaying seeking hearing care, which leads to delayed diagnoses and rehabilitative interventions. Therefore, these misperceptions must be addressed. Misperceptions could be modified through interventions to increase knowledge and awareness related to hearing loss and hearing technology.^4950 69^,^72^

There are cultural and regional differences in perceptions of hearing loss and hearing technology,^38 40^ and these perceptions are likely to change over time. There may be other factors (not discussed in this review) that contribute to differences in perceptions. Future research should focus on understanding those factors to create tailored messaging and interventions to modify misperceptions related to hearing loss and hearing technology. Research could also clarify differences in perceptions towards subgroups of people with hearing loss and/or who use different forms of hearing technology (eg, air or bone conduction hearing aids, cochlear implants), topics which are not covered in this review.

### Strengths and limitations

To the authors’ knowledge, this article provides the first comprehensive review of perceptions of hearing loss and hearing technology among members of the general public and healthcare providers. This scoping review captures the strengths and limitations of each study. We did not conduct a risk of bias assessment given the nature of this scoping review. Because the scope of this review was relatively broad, there were few studies on certain topics (eg, perceptions on the impact of hearing loss compared with other conditions). For articles published in Spanish, a single author identified articles, using handsearching, and extracted data. This decision was made based on the languages that the members of the study team speak fluently. Although it is possible that this process could introduce errors, all members of the study team agreed on the inclusion and exclusion of each article included in the review. There may be articles published in other languages (in addition to English and Spanish) that were not identified.

## Conclusions

Results from this scoping review indicate perceptions of hearing loss and hearing technology are varied but often misinformed and negative. Improved understanding of these perceptions can inform context-appropriate initiatives to raise awareness and change behaviours informed by those perceptions. Such initiatives could reduce misperceptions towards individuals with hearing loss, and ultimately, encourage individuals with hearing loss to seek hearing care in a timely and appropriate manner.

## supplementary material

10.1136/bmjph-2024-001187 online supplemental file 1

10.1136/bmjph-2024-001187 online supplemental figure 1

## Data Availability

Data sharing not applicable as no datasets generated and/or analysed for this study.
