# Supplementary material for: Perceptions of hearing loss and hearing technology among the general public and healthcare providers: a scoping review
Source: BMJ Public Health. 2024 Oct 15;2(2):e001187. doi: 10.1136/bmjph-2024-001187 (PMC11816092; doi:10.1136/bmjph-2024-001187)
Supplement: online supplemental file 1 [file bmjph-2-2-s001.pdf]

Online supplemental file 1: Search strings for each database and details on grey literature search. Searches were conducted August 28, 2023.

Databases

1. PubMed

Advanced search

n=115

Filters: English

("hearing loss"[Title/Abstract] OR "hearing impair*"[Title/Abstract] OR deaf*[Title/Abstract] OR "hearing problems"[Title/Abstract]) AND (perceptions[Title/Abstract] OR awareness[Title/Abstract] OR "knowledge, attitudes, and beliefs"[Title/Abstract] OR "social representation") AND ("general public"[Title/Abstract] OR "general population"[Title/Abstract] OR "health care provider"[Title/Abstract] OR "healthcare provider"[Title/Abstract] OR "health care worker"[Title/Abstract] OR "healthcare worker"[Title/Abstract] OR doctor[Title/Abstract] OR physician[Title/Abstract] OR nurse[Title/Abstract]])

2. Scopus

Advanced search

n=250

Filters: English

(ABS (({hearing loss} OR {hearing impair*} OR deaf* OR {hearing problems})) AND ABS ((perceptions OR awareness OR {knowledge, attitudes, and beliefs} OR {social representation})) AND ABS (({general public} OR {general population} OR {health care

provider} OR {healthcare provider} OR {health care worker} OR {healthcare worker} OR doctor OR physician OR nurse))) AND (LIMIT-TO (LANGUAGE , "English"))

3. Ovid Medline

Advanced search

n=135

Filters: English

("hearing loss" OR "hearing impair$" OR deaf$ OR "hearing problems") AND (perceptions OR awareness OR "knowledge, attitudes, and beliefs" OR "social representation") AND ("general public" OR "general population" OR "health care provider" OR "healthcare provider" OR "health care worker" OR "healthcare worker" OR doctor OR physician OR nurse)

Grey literature

Grey literature sources included white papers, newsletters, reports, proceedings, dissertations, theses, or published abstracts or conference papers. Sources were identified using the keywords (or derivations of) “hearing loss,” “deafness,” “perceptions,” and “attitudes” using Google Scholar and other sources.
