# Supplementary material for: Perceptions of hearing loss and hearing technology among the general public and healthcare providers: a scoping review
Source: BMJ Public Health. 2024 Oct 15;2(2):e001187. doi: 10.1136/bmjph-2024-001187 (PMC11816092; doi:10.1136/bmjph-2024-001187)
Supplement: online supplemental figure 1 [file bmjph-2-2-s002.pdf]

Supplementary Figure 1: Flow diagram (PRISMA 2020) summarizing the article screening and selection process.

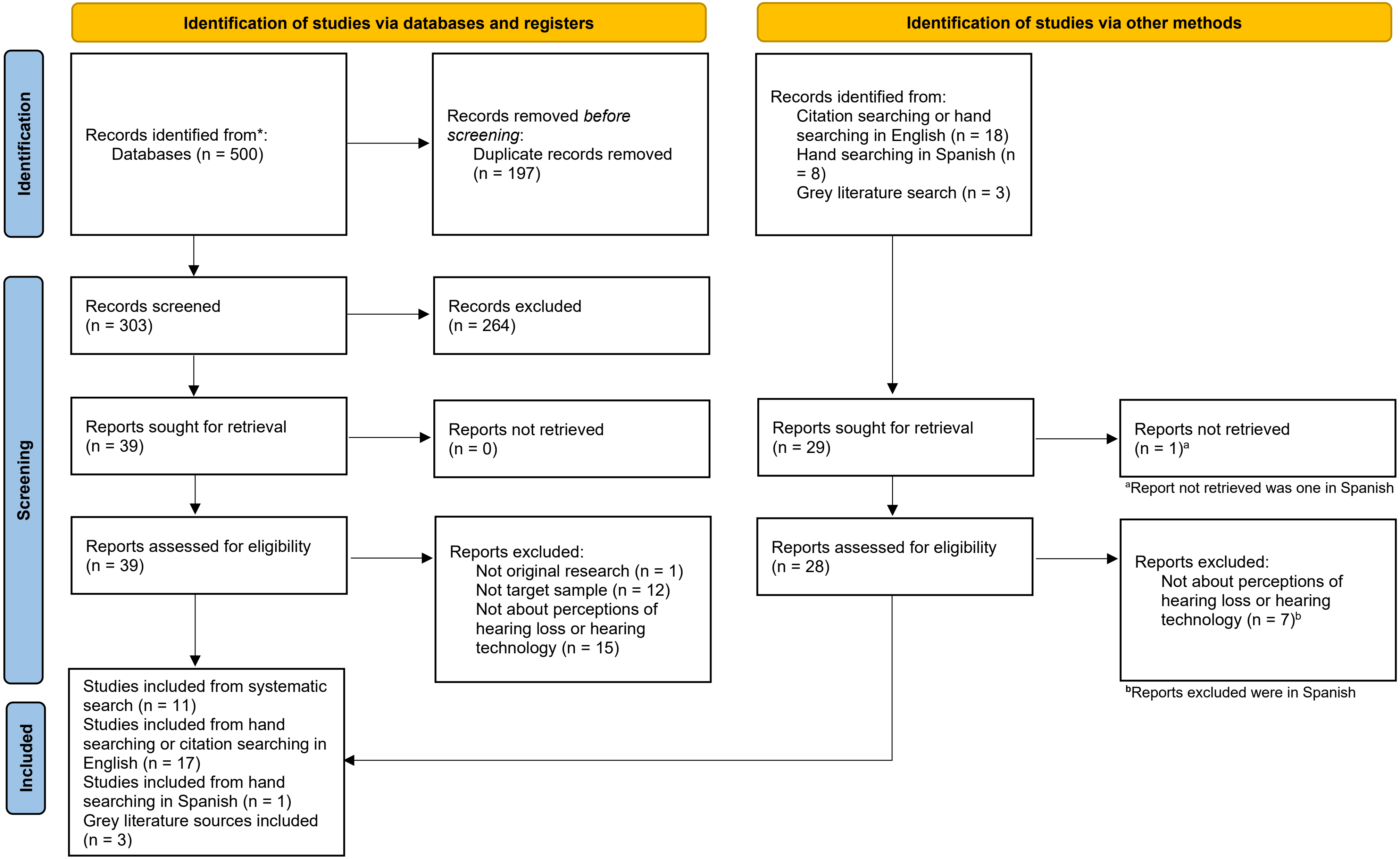

From: Page MJ, McKenzie JE, Bossuyt PM, Boutron I, Hoffmann TC, Mulrow CD, et al. The PRISMA 2020 statement: an updated guideline for reporting systematic reviews. BMJ 2021;372:n71. doi: 10.1136/bmj.n71. For more information, visit: http://www.prisma-statement.org/
